# Metabolic pathways synthesis based on ant colony optimization

**DOI:** 10.1038/s41598-018-34454-z

**Published:** 2018-11-06

**Authors:** Matias F. Gerard, Georgina Stegmayer, Diego H. Milone

**Affiliations:** Research Institute for Signals, Systems and Computational Intelligence (sınc(i)), FICH–UNL/CONICET, Ciudad Universitaria UNL, (S3000), Santa Fe, Argentina

## Abstract

One of the current challenges in bioinformatics is to discover new ways to transform a set of compounds into specific products. The usual approach is finding the reactions to synthesize a particular product, from a given substrate, by means of classical searching algorithms. However, they have three main limitations: difficulty in handling large amounts of reactions and compounds; absence of a step that verifies the availability of substrates; and inability to find branched pathways. We present here a novel bio-inspired algorithm for synthesizing linear and branched metabolic pathways. It allows relating several compounds simultaneously, ensuring the availability of substrates for every reaction in the solution. Comparisons with classical searching algorithms and other recent metaheuristic approaches show clear advantages of this proposal, fully recovering well-known pathways. Furthermore, solutions found can be analyzed in a simple way through graphical representations on the web.

## Introduction

Nowadays, information of metabolic pathways for a large number of living beings is available in databases such as KEGG^1^, MetaCyc^2^ and Brenda^3^. This allows the online exploration of the enzymes, biochemical reactions catalyzed, and the involved substrates and products. Although individual rules for producing compounds are well-known, it is still a challenge to identify the adequate sequence of reactions required for the synthesis of several compounds as part of a (novel) complex metabolic network with several branches^4^.

Traditionally, metabolic pathway synthesis of a target compound from a given source has been addressed by methods based on graphs. The main reason is to avoid shortcomings of stoichiometric approaches when applied to networks of large size^5^. The first step is to model compounds and reactions as an appropriate graph^6^. In a general approach for modeling, nodes indicate compounds and edges link substrates and products of the same reaction. The next step is searching for a path over the graph, that connects the source with the target compound using some search method. These methods were based mostly on classical Breadth-First Search (BFS) and Depth-First Search (DFS) algorithms^7^. The main problem faced by these methods is avoiding the commonly called *pool compounds*, such as ATP, NAD and water, which are involved in many different reactions carrying out several tasks. Since they have a high connectivity degree, pool compounds are frequently included as intermediate in the solutions found, producing biologically unfeasible pathways.

A systematic approach to deal with pool compounds consists in describing their structures in terms of features vectors^8^ or fingerprints^9^. These representations can be used, in combination with a similarity measure, to select the next more similar compound to the current one, thus avoiding pool compounds. Another option is assigning a cost to nodes or links of the graph according to the number of reactions in which each compound participates, and then search for pathways with the lower costs^10,11^. Kotera and co-workers have manually characterized each substrate-product pair on every known reaction according to the fulfilled function^12,13^. Using this characterization, several methods first build graphs without biologically irrelevant connections, and then search for metabolic pathways taking into account only those pairs describing main functions^14,15^. The number of atoms shared between substrates and products of reactions has also been used to avoid pool compounds. Based on this information, some methods search for metabolic pathways that maximize the number of atoms transferred from the source to the target compound^16^, or at least preserve a given number of them in the path^17^. An improved version of this approach assigns a cost to the connections in the graph based on structural similarity of the compounds and the thermodynamics of the reaction that involves them^18^. Khosraviani *et al*. have proposed an AND/OR boolean representation of the graph using matrix notation^19^. It allowed search for pathways between source and target compounds over a reduced search space by applying boolean operations. However, these strategies have the limitation of finding solutions only as linear sequences of reactions (or a combination of them), and they do not take into account the availability of compounds already synthesized. In many cases, this leads to the synthesis of metabolic pathways in an uncoordinated fashion, providing solutions without biological sense.

In a previous work, we proposed an algorithm called Evolutionary Metabolic Seeker (EvoMS), which synthesized metabolic pathways using information on the availability of compounds^20,21^. EvoMS models a metabolic pathway as a sequence of feasible reactions that start from a set of initial substrates. In this tool, we proposed the *set-of-compounds* (SoC) model, where a set containing substrates for all reactions in a given sequence is iteratively updated with the products of each new feasible reaction added to the sequence. Thereby, searching for a pathway consist in finding a sequence of feasible reactions that relates a given group of compounds, subject to the availability of initial substrates. However, EvoMS is unable to preserve a set of feasible solutions along the search, since the availability of substrates is not guaranteed when intermediate solutions are combined to produce new ones. As a consequence, the search is very slow and the synthesis of pathways that relate multiple compounds in large search spaces is not always possible.

**Figure 1:**
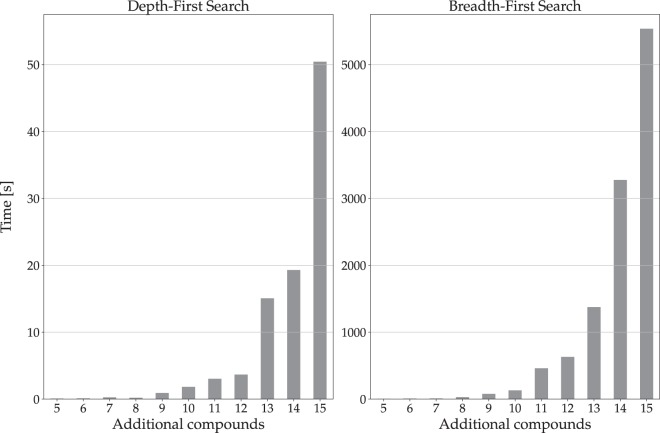
Average time required (100 runs) for DFS (left) and BFS (right) to search for a pathway between 2 compounds. The *x*-axis indicates the number of compounds added to the minimum required set.

Synthesizing metabolic pathways has the challenge of exploring a large solution space, which grows when more reactions and compounds are involved. When the SoC model is considered, this problem becomes more difficult and clearly imposes a limitation on the use of classical DFS- and BFS-based algorithms. As frequently happens in real problems, the minimum set of available compounds required for synthesizing a pathway may be not completely known in advance. In consequence, more compounds than necessary are generally added to the initial set, trying to prevent losing potential solutions because some substrates are not available. In order to evaluate how the performance of the algorithms behaves in this context, we designed an experiment where we knew in advance the solution to be found and the minimum set of compounds needed to synthesize it. For this purpose, a list of 79 reactions belonging to the glycolysis was extracted from KEGG, and used to synthesize a pathway to produce 2-phospho-D-glycerate from D-glucose-1-phosphate. Furthermore, the minimum set of compounds required to find the solution was identified. Then, we systematically added a larger number of compounds to increase the size of the set of initially available ones, and run BFS and DFS to explore the search space generated by the SoC model for each initial set of compounds. Figure 1 shows the growth of searching time (average over 100 runs) for BFS and DFS algorithms according to the number of compounds added to the minimum set of initially available ones. As it can be expected, average time grows exponentially with the increase in the size of the available compounds set.

A bio-inspired metaheuristic that has proved to efficiently solve such large graph-based problems is the ant colony optimization algorithm (ACO). The ACO is an important technique in the field of Swarm Intelligence, and it is inspired on the behavior of real ant colonies searching for food^22^. The ants deposit pheromone on the ground in order to mark the routes, from the nest to food, which should be followed by other members of the colony. Accumulation of pheromones over paths along the iterations favor solutions that minimize a cost function^23^. Those algorithms have been successfully applied to a wide range of problems in many different areas^24^. Particularly, they have proved to be a powerful tool solving biological problems related to protein folding^25^, genetic interactions detection^26^, RNA sequence design^27^, protein-protein interaction inhibitors design^28^, protein structure optimization^29^ and protein-ligand docking^30^. Moreover, they have outperformed genetic algorithms in a wide range of combinatorial optimization problems^31–35^.

In this work we propose a novel ant-based algorithm to synthesize metabolic pathways, to efficiently explore large search spaces of reactions. Our proposal takes advantage of the way on which ants perform the exploration to incorporate information of the compounds availability, in order to build feasible solutions. Furthermore, since this algorithm uses the SoC model to search, it is possible to find solutions with both linear and branched topology. This algorithm can be suitable for applications such as synthetic biology, interpretation of metabolomics experiments and gap filling in metabolic reconstructions.

## Proposed Computational Method

### State space model and metabolic pathways

Metabolic pathways are networks built by compounds and the biochemical reactions that relate them. These reactions allow the synthesis of new compounds from other ones. Formally, the reactions are described by typical chemical equations as *S*(*r*) ↔ *P*(*r*), where *S*(*r*) and *P*(*r*) correspond to the substrates and the products, respectively^36^. Then, metabolic pathways can be described as sequences of sets of compounds (substrates for next reactions), with composition and size defined by the order on which the reactions of the pathway are performed^37^. Following this reasoning, the state space for the problem of synthesizing metabolic pathways can be build considering each state as a set of compounds and the relations among them. Then, transitions between states are given by those reactions that can be carried out with the available substrates in the current state. It must be noted that while available connections among compounds are known and fixed for a given set of reactions (typical compounds-and-reactions graph), the graph describing the state space changes according to the initial set of available compounds specified. Furthermore, the number of nodes is even larger than for compounds-and-reactions graphs, since each node represents a unique state, which in turn corresponds to a metabolic network in itself (set of compounds and the relations among them). As a result, every path in the search tree built to find a solution on this graph will be feasible, because reactions for which substrates are available in the current node can only be performed.

**Figure 2:**
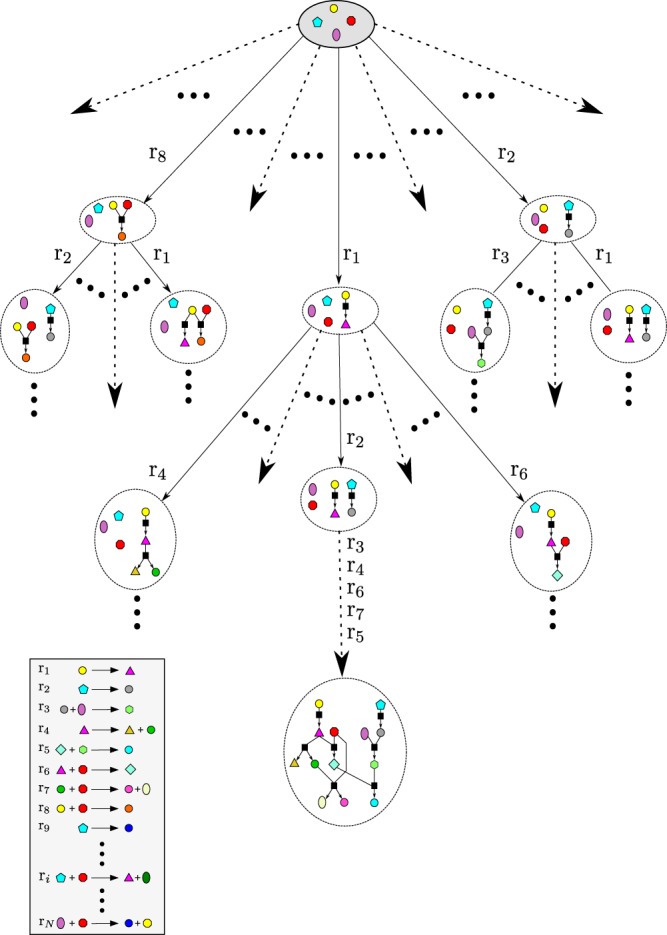
Example of a tree for the exploration of the state space.

Figure 2 shows an example of a typical tree to explore the search space. The root node, composed by a set of four compounds without links, is the initial state of the search. When reaction *r*_1_ is applied, for example, a new state with one link and five compounds (triangle added) is reached. Now, applying reaction *r*_2_ over this node, we can reach a new one with a new link and an additional compound. Following this strategy we can reach to a final state describing a metabolic pathway that relates 13 compounds (bottom of the figure).

It is important to highlight here that this approach is clearly different from graph search methods^37^, because our proposal does not build first the complete graph and then performs the search within it. Instead, metabolic pathways are grown step by step, by choosing one feasible reaction at a time from a list of available reactions. Then, the chosen reaction is added to a sequence that starts from a set of available compounds. Moreover, choosing reactions is done with a given probability which is learned while solutions are synthesized. Finally, each state explored during the process corresponds to a complete metabolic pathway and not just to a single compound, as typically occurs in with classical searching methods.

### Ant-based algorithm for searching metabolic pathways

Table 1 presents the general steps of the ant-based method which we called PhDSeeker (Pheromone-Directed Seeker). The algorithm receives four inputs: a set ${\mathscr{D}}$ of compounds to relate; a subset ${\mathcal I} \subseteq {\mathscr{D}}$ of compounds which can be used as initial substrate for the metabolic pathway; a list ${\mathcal R}$ of reactions that can be used to build the solution; a set ${\mathscr{C}}$ of freely available compounds (such as water, ATP, etc). As output, it returns a list of reactions ${{\boldsymbol{\pi }}}^{\ast }=[{\pi }_{0}^{\ast },\cdots ,{\pi }_{i}^{\ast },\cdots ,{\pi }_{N}^{\ast }]$ describing the best feasible metabolic pathway found from the initial conditions specified.

**Table 1:**
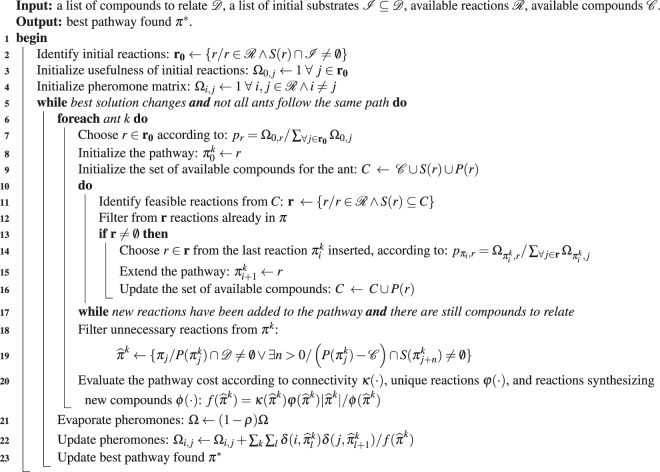
Ant-based synthesis of metabolic pathways.

PhDSeeker starts building the list of reactions ***r***_0_ that use any compound in ${\mathcal I}$ as substrate. From those reactions, each ant will choose one as the initial reaction of its path. Next, the pheromone matrix **Ω** is initialized setting Ω_*i*,*j*_ = 1. This is used by ants along the search to store the frequency of reactions *i* → *j* in the solutions. For the particular case of *i* = 0, **Ω** stores information of the usefulness for the reactions in ***r***_0_. This value indicates how frequently a given initial reaction leads to a solution.

The searching process begins after **r**_0_ and **Ω** have been initialized. On each iteration of the algorithm, every ant performs an independent search, following the same steps (lines 7 to 19). Initially, the ant *k* chooses one reaction *r* from ***r***_0_ according to its probability *p*_*r*_, which depends on the values stored in the first row of **Ω** (line 8). Then, it is used to set the first reaction ${\pi }_{0}^{k}$ of the pathway ***π***^*k*^. In addition, substrates *S*(*π*_0_) and products *P*(*π*_0_) of this reaction are combined with the available compounds ${\mathscr{C}}$ to build the initial set of compounds *C*^*k*^ that the ant uses to synthesize a pathway linking compounds in ${\mathscr{D}}$ (line 9).

After the first reaction is inserted into the pathway, the ant repeat five operations (lines 10 to 17). Initially, the ant identifies all the reactions **r** for which the substrates are in *C*^*k*^, filtering those that are already in the pathway ***π***^*k*^. Then, it chooses one reaction *r* ∈ **r** according to its probability ${p}_{{\pi }_{i},r}$, and adds the selected reaction to the pathway (lines 14-15). Finally, the ant updates its set of available compounds *C*^*k*^ with products of the selected reaction (line 16). These operations are repeated by every ant until there are no more feasible reactions to synthesize the pathway (**r** = ∅), or a metabolic pathway synthesizing all the final products (${\mathscr{D}}\subseteq C$), is found.

Once the ant completed the search, unnecessary reactions are removed of ***π***^*k*^, and the cost of the resulting pathway ${\hat{\pi }}^{k}$ is calculated (line 18). The pathway cleaning step consists in discarding reactions that do not synthesize any of the compounds in ${\mathscr{D}}$, that is, the ones that only produce compounds belonging to ${\mathscr{C}}$, or those which synthesize compounds that are not substrate for any reaction. The cost $f(\hat{{\boldsymbol{\pi }}})$ is calculated based on the evaluation of four characteristics of the pathway (line 19): number of reactions in the pathway $|\hat{{\boldsymbol{\pi }}}|$; number of unique reactions $\varphi (\hat{{\boldsymbol{\pi }}})$; number of reactions synthesizing new compounds $\phi (\hat{{\boldsymbol{\pi }}})$; and connectivity of the pathway $\kappa (\hat{{\boldsymbol{\pi }}})$. The number of unique reactions is calculated as $\phi (\hat{\pi })=|\{{\hat{\pi }}_{i}/{\hat{\pi }}_{i}\ne {\hat{\pi }}_{j},\forall j < i\}|$, and penalize solutions including reactions used with both directions. The number of reactions synthesizing new compounds is determined as $\varphi (\hat{\pi })=|\{{\hat{\pi }}_{i}/\{P({\hat{\pi }}_{i})-\{{\cup }_{\forall j < i}P({\hat{\pi }}_{j})\}-{\mathscr{C}}\}\ne \varnothing \}|$, where $P({\hat{\pi }}_{i})$ is the set of products for reaction ${\hat{\pi }}_{i}$. This measure reaches its maximum value when all reactions in the pathway produce at least one new compound, not previously synthesized. Connectivity evaluates the number of final products (compounds in ${\mathscr{D}}$ without the initial substrate) synthesized from the initial substrate in the pathway. Let ${X}_{0}=\{S({\hat{\pi }}_{0})\cap {\mathscr{D}}\}$ be an initial set of compounds containing only the initial substrate used by the first reaction of the pathway. The update of this set is performed according to

1 $${X}_{i+1}=\{\begin{array}{cc}{X}_{i}\cup (P({\hat{\pi }}_{i+1})-{\mathscr{C}}) & {\rm{i}}{\rm{f}}\,{X}_{i}\cap S({\hat{\pi }}_{i+1})\ne \varnothing ,\\ {X}_{i} & {\rm{i}}{\rm{n}}\,{\rm{o}}{\rm{t}}{\rm{h}}{\rm{e}}{\rm{r}}\,{\rm{c}}{\rm{a}}{\rm{s}}{\rm{e}}.\end{array}$$

The latest updated set *X*_*N*_ contains all the compounds synthesized by any reaction related to the initial compound. Based on this set, connectivity $\kappa (\hat{{\boldsymbol{\pi }}})$ can take value

2 $$\kappa (\hat{\pi })=\{\begin{array}{cc}1 & {\rm{i}}{\rm{f}}\,|{X}_{N}\cap {\mathscr{D}}|/|{\mathscr{D}}|=1,\\ \alpha  & {\rm{i}}{\rm{n}}\,{\rm{o}}{\rm{t}}{\rm{h}}{\rm{e}}{\rm{r}}\,{\rm{c}}{\rm{a}}{\rm{s}}{\rm{e}},\end{array}$$

being *α* a constant that establishes the cost difference between partial solutions (only some final products are synthesized from the initial substrate) and complete ones. Therefore, when $\alpha \ll 1.0$ the solutions that relate only some of the compounds will cost less than those that relate them all. In contrast, when $\alpha \gg 1.0$, the solutions that link all the compounds will have lower cost, and will be the ones that the ants will try to build. A recommended value is *α* = 10*N*_*k*_, being *N*_*k*_ the number of ants used in the search (see Supplementary Figure S2 for a detail on the effect of *α* for more details).

After ants have removed unnecessary reactions from the pathways and the cost of each solution was evaluated, the pheromone matrix is updated following two mechanisms (lines 20 and 21). First, the pheromone evaporation is done by removing a proportion *ρ* of the pheromones, in order to emulate the natural process of loss of information associated to evaporation. Next, the elements of **Ω** are updated according to the reactions used in the pathways found, and the cost of the solutions. Thus, given a pathway ${\hat{\pi }}^{k}$, the usefulness of the first reaction ${\hat{\pi }}_{0}^{k}$ is updated by adding the quantity 1/*f*(${\hat{\pi }}^{k}$) to ${{\rm{\Omega }}}_{0,{\hat{\pi }}_{0}^{k}}$. Then, the reactions sequence of the pathway is traversed, and the pheromone value ${{\rm{\Omega }}}_{{\hat{\pi }}_{i}^{k},{\hat{\pi }}_{i+1}^{k}}$ corresponding to every couple ${\hat{\pi }}_{i}^{k},{\hat{\pi }}_{i+1}^{k}$ is updated by adding the quantity 1/*f*(${\hat{\pi }}^{k}$). Once the three steps of collective knowledge update are finished, the best solution found is saved in π^*^.

The algorithm searches until the best solution does not change for a given number of iterations, and all the ants follow different paths according to their costs.

## Datasets and Measures

Reactions used in the experiments were extracted from the KEGG database^1^ (other repositories such as MetaCyc^38^ could be used as well). The direction for each reaction was assigned using the information contained in the KGML files associated to the reference maps^39,40^. Each reversible reaction was modelled as a pair of independent reactions with opposite direction. A total of 5 datasets of reactions (*glycolysis*, *proline*, *xproline*, *multipaths*, *ecoli*) were used in the experiments. Details on the datasets of reactions and the list of freely available compounds, are provided in the Supplementary Material, Tables S1 and S2.

Algorithms were evaluated on searching time *t*, the number of reactions *N*_*R*_ in the solution and the branching factor *β*. Even though searching time depends on many elements, it was used as a rough indicator of computational cost. The branching factor evaluates the relation among reactions in the pathway, measuring the average number of reactions that use every non-abundant substrate. It is calculated according to

3 $$\beta ({\boldsymbol{\pi }})=\frac{1}{|{S}_{f}^{\ast }|}\sum _{i=1}^{|{S}_{f}^{\ast }|}\sum _{j=1}^{|{\boldsymbol{\pi }}|}\,{{\bf{1}}}_{{s}_{i}\subseteq S({r}_{j})},$$

where ${S}_{f}^{\ast }$ are the substrates of all reactions in **π** after filtering the abundant compounds, |***π***| is the pathway size, **1** is the indicator function, *s*_*i*_ is the *i*- th compound of ${S}_{f}^{\ast }$, and *S*(*r*_*j*_) are the substrates of reaction *r*_*j*_.

For comparisons with other state-of-the-art methods, a benchmark dataset of 42 reference pathways derived from the aMAZE database^41^ and provided by Huang *et al*.^18^ have been used. It consists of real pathways up to 10 reactions belonging to *E. coli*, *S. cerevisiae*, and *H. sapiens* that are commonly used for evaluation of pathfinding methods in the literature^18^. Performance on the available synthesized pathways was evaluated according to measures defined in literature^17^, being: true positives (TP) those elements (compounds or reactions) found in both the reference and the synthesized pathway; false positives (FP) those elements in the synthesized pathway but not in the reference one; and false negatives (FN) correspond to elements in the reference pathway but not in the synthesized one. Precision is calculated as PR = TP/(TP + FP) and, in this context, it provides information about the proportion of compounds/reactions in the synthesized pathway which effectively are in the reference one. The higher this value, the fewer compounds/reactions outside the reference pathway will be part of the synthesized pathway. Recall is calculated as RC = TP/(TP + FN), and indicates the proportion of compounds/reactions of the reference pathway that are in the synthesized one (proportion of the reference pathway effectively recovered). Accuracy is calculated as Acc = (PR + RC)/2, and gives a balance between both previous measures.

## Results and Discussion

### Comparison of searching times

Searching time required for DFS, BFS, EvoMS and PhDSeeker for finding metabolic pathways was evaluated in a simple problem. This consisted on the search of a metabolic pathway between compounds C00103 (D-glucose-1-phosphate) and C00631 (2-phospho-D-glycerate), using the *glycolysis* dataset of reactions and C00103 as initial substrate. For this experiment, the initial set of available compounds containing only those required for finding at least one solution was built combining freely available compounds with substrates of reactions using C00103. However, it is important to remark that the initial set of available compounds can be built with as many compounds as it is desired. A reasonable starting point could include compounds such as water, NADH, H^+^ and many others that are actually freely available in living organisms (see Supplementary Table S2 for an example). Then, this set could be extended with other compounds based on the knowledge of the organism. For example, metabolomic information could be used to identify some extra compounds that could be included in the set of available ones.

In order to generate different initial conditions for the searching problem, an increasing number of extra compounds, randomly selected, was added to the initial set of available compounds. It should be noted that the minimum set of initially available compounds can be specified for this problem, since the solution is well-known. We performed 16 experiments, each one with 100 runs. Search operators (reactions) were randomly sorted on each run of the BFS and DFS algorithms. The maximum search depth for DFS was 10, corresponding to twice the number of reactions of the shortest metabolic pathway. Preliminary experiments indicated that EvoMS required up to 100 individuals to find a solution to this problem. In the case of PhDSeeker, it was observed that 5 ants were enough to build a metabolic pathway linking both compounds.

**Figure 3:**
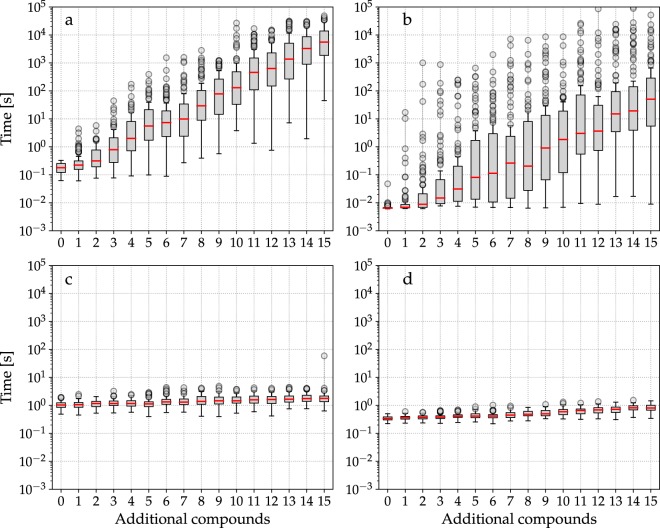
Searching times required to find a metabolic pathway considering a growing number of compounds added to the minimum set of available ones. (**a**) BFS; (**b**) DFS; (**c**) EvoMS; (**d**) PhDSeeker. Red line denotes the median, and circles indicate outliers.

Figure 3 shows a boxplot of the searching time for all methods. As it can be seen, classical methods show an exponential increase in the searching time, from tenths of seconds to minutes, when a large number of extra compounds is taken into account. Since the root node corresponds to the set of initially available compounds, feasible reactions from this set are the possible branches for the first level of the search tree. In consequence, the increase in the size of this initial set is quickly reflected as an increase in the number of branches for the first level of the search tree, and this effect is then translated to the following levels. Clearly, it leads to a growth in the number of states to be explored to find a solution. Moreover, it can also be appreciated a high variability in the searching time and large number of outliers, because there is not *a-priori* knowledge about how the search operators should be applied to find solutions in the minimum number of steps. In contrast, searching time for metaheuristic algorithms are practically not modified when increasing the number of extra compounds, because they perform a smartest exploration of the search space. Furthermore, this makes searching times variability very small, staying always around the second.

Clearly, this result shows that performance of classic search methods is strongly influenced by the initial conditions of the problem. Even in this simple problem with a relatively small search space, the searching time easily becomes unmanageable. Instead, the effect on metaheuristics is minimal, making them a suitable tool to address real problems of higher complexity.

### Increasing the search space

Performance of EvoMS and PhDSeeker was compared by searching pathways among compounds C00025 (glutamate), C00122 (fumarate) and C00763 (proline) in *proline* and *xproline* datasets of reactions. Solution is well-known for *proline* dataset of reactions, and corresponds to a branched pathway starting from C00025. We expected that both algorithms be able to find the solution, regardless the size of the search space.

Experiments were performed with the following configuration. EvoMS was run with *N*_*k*_ = 100 individuals, crossover probability *p*_*x*_ = 0.8, mutation probability *p*_*m*_ = 0.08, erasure probability *p*_*e*_ = 0.8 and valid insertion probability *p*_*v*_ = 0.5. Those parameters were determined in previous experiments^21^, specifying the maximum number of generations to *G*_*M*_ = 1000. In all cases, the best individual was preserved on each generation (elitism) and a generational gap of 30 individuals was used. PhDSeeker was run up to a maximum of 100 iterations, using *N*_*k*_ = 10 ants and an evaporation rate of *ρ* = 0.1. In a preliminary experiment with a completely independent dataset and reference pathway it was observed that those parameters provide a good performance (see Supplementary Figure S2 for more details). For both algorithms, the number *N*_*k*_ of individuals was selected to be the minimum number of individuals required for finding a metabolic pathway linking the specified compounds.

Table 2 shows performance measures evaluated in both datasets of reactions. While both algorithms generate solutions in a wide range of sizes, metabolic pathways found by EvoMS have significantly more reactions than the pathways found by PhDSeeker (*p* < 0.001, with the Wilcoxon signed-rank test), when considering the *proline* dataset of reactions. This is due to the presence of a greater number of redundant reactions that are not filtered in the solutions. Regarding the branching factor, it must be noted that both algorithms have *β* > 1.0, indicating that, in fact, solutions are branched. Difference in the average value are given by the way in which each method initializes the pathway search. In PhDSeeker, only one reaction using the initial substrate is allowed; since only substrates, for this reaction, are provided together with the available compounds. It makes that the branching factor depends exclusively on the branches in the pathway found. Instead, EvoMS builds the set of available compounds taking into account substrates for all the reactions using the initial substrate. It makes feasible the incorporation of several reactions that depend on the initial substrate. Thus, the branching factor will be increased by the presence of these additional initial reactions. Concerning the searching time, results show that the practical performance of both algorithms is comparable in general terms. Summarizing, we can say that results obtained with both algorithms become more similar when increasing the size of the search space.

**Table 2:** Average performance for 3-compounds (100 runs of each method).

	*proline*		*xproline*	
	EvoMS	PhDSeeker	EvoMS	PhDSeeker
N_*R*_	13.99 (3.85)	9.80 (0.81)	8.20 (3.34)	6.99 (2.69)
*β*	1.47 (0.18)	1.27 (0.08)	1.45 (0.28)	1.24 (0.08)
*t*	13.31 (7.44)	1.90 (0.38)	5.01 (3.02)	6.18 (1.67)

N_*R*_: Number of reactions comprising the solution. *β*: branching factor (average number of reactions that use every non-abundant substrate). *t*: time required to find a solution. Standard deviation in brackets.

### Linking more compounds

Results of the previous section shown that both metaheuristics find pathways that relate the specified compounds in search spaces of different sizes. In this section, we compared the capability for searching pathways in situations where several compounds can be used as starting substrate. Experiments were performed using the *multipaths* dataset of reactions, searching for pathways that relate compounds C00036 (oxaloacetate), C00118 (glyceraldehyde-3P), C00181 (D-xylose) and C00267 (*α*-D-glucose). Using these compounds as initial substrate, four experiments (100 runs each) were done. Additionally, we also performed experiments using the automatic initialization strategy to select the best initial substrate. In all cases, we analyzed the proportion of runs where a metabolic pathway linking the four compounds was found. EvoMS and PhDSeeker were run with similar experimental configuration used in the previous section.

**Figure 4:**
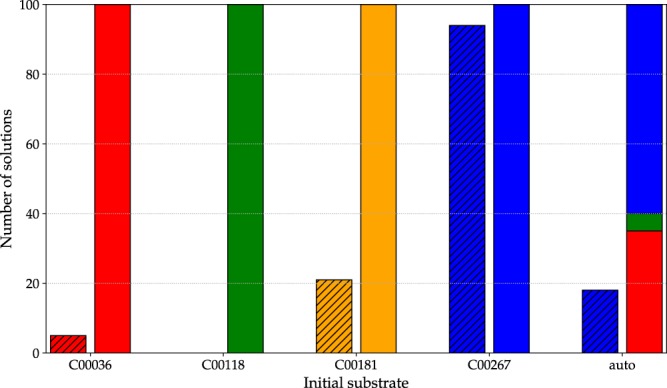
Number of runs in which a solution was found.

Figure 4 shows the number of runs where a metabolic pathway was found. Every couple of bars presents results for EvoMS (striped bars) and PhDSeeker using a given initial substrate. As it can be seen, EvoMS is only able to find solutions only when C00267 is used as initial substrate. Furthermore, this algorithm is unable to find any solution using C00118. Instead, PhDSeeker founds solutions in every run, regardless of the initial substrate considered. Moreover, the automatic selection of initial substrate used three of the four compounds for finding pathways to relate them. These results indicate that PhDSeeker outperformed EvoMS for searching pathways linking several compounds. The ant-based algorithm found a solution on each run, regardless of the mechanism used to select the initial substrate.

### Comparison with state-of-the-art algorithms

Performance of PhDSeeker was compared with several state-of-the-art metabolic pathfinding methods included in a very recent review^42^. Based on the availability of the algorithms, the comparison was made using AGPathFinder (search based on group-of-atoms-tracking and thermodinamics)^18^, LPAT (search based on maximization of atoms transferred from source to target)^17^, FMM (search based on the minimization of the number of known pathways to be combined in the solution)^43^ and RouteSearch (search based on atom-tracking and thermodinamics)^44^. Furthermore, in order to extend the comparison to other approaches, we also include two methods for subgraph extraction (see Table 3): Graphtools^11^ and SubNet^45^ (using *k Walks* strategy). For each pathfinding method, the first 10 solutions for each real reference pathway were evaluated, and the solution with higher accuracy was chosen to calculate performance measures. In case of subgraph extraction methods, each network found was taken as solution. Clearly, results shown in Table 3 are similar for measures calculated on compounds or reactions. Precision results indicate that pathways recovered by PhDSeeker are composed mainly by elements of the reference pathways, incorporating only very few foreign components. Recall values show that a high proportion of the reference pathways is recovered by PhDSeeker, being only FMM slightly better in terms of compounds. However, it is important to note that although compounds for FMM are mostly the same than in the reference pathways, this is not the case for reactions, since it does not use the same reactions as in the reference pathways. Regarding accuracy, PhDSeeker has the highest values for both compounds and reactions, indicating that it can achieve the best balance between Precision and Recall: pathways mainly contain elements of the reference pathway, and only a few external elements are included in some of the solutions.

**Table 3:** Comparison of performance between PhDSeeker and several state-of-the-art methods.

	COMPOUNDS						
	AGPathFinder	LPAT	FMM	RouteSearch	Graphtools	SubNet	PhDSeeker
Precision	0.866	0.873	0.887	0.822	0.927	0.457	**0**.**958**
Recall	0.826	0.872	**0**.**926**	0.818	0.836	0.678	0.914
Accuracy	0.846	0.873	0.907	0.820	0.881	0.568	**0**.**936**
	**REACTIONS**						
	**AGPathFinder**	**LPAT**	**FMM**	**RouteSearch**	**Graphtools**	**SubNet**	**PhDSeeker**
Precision	0.648	0.777	0.875	0.662	0.712	0.160	**0**.**883**
Recall	0.629	0.841	0.840	0.690	0.681	0.584	**0**.**861**
Accuracy	0.638	0.809	0.857	0.676	0.697	0.372	**0**.**872**

Best results in bold.

Furthermore, it must be highlighted that our algorithm is designed for finding the shortest feasible pathways. In consequence, PhDSeeker was capable of finding solutions that share reactions with the reference pathways and that are also shorter, because it replaced several reactions by a unique step in order to minimize the pathway cost. While this may reduce precision and recall, pathways found are still fully feasible. Finally, it is important to note that our proposal has achieved these good results by using a simple model, which does not need to use information of the structure of compounds nor the thermodynamics of reactions.

### Metabolic pathways in a model organism

#### Validation using a standard pathway

Due to the fact that the most important point is the biological significance of results, we have used here the real well-known pathway for the synthesis of L-lysine (C00047), L-methionine (C00073) and L-threonine (C00188) from oxaloacetate (C00036), performed in *E. coli*, to evaluate the feasibility of solutions found with PhDSeeker. The algorithm was run using 10 ants, being the number of reactions in the *ecoli* dataset the boundary specified for the search.

Experimental results show that both pathways were similar in most reactions, having only a small difference in the mechanism for synthesizing C00073. While the standard pathway uses reactions R03260 and R01286 to transform C01118 into C00155, the solution found with PhDSeeker only requires reaction R01288 to perform this transformation. It is important to highlight that reactions R03260 and R01288 are catalyzed by the same enzyme (EC 2.5.1.48), but differ in the substrates used. This indicates that C00155 can be produced by means of the two-step way when substrates for the one-step transformation are not available. In conclusion, from a biological point of view, both pathways are similar. A simple representation of both pathways, the standard and the solution found by PhDSeeker, is provided in Supplementary Material, Figure S1.

#### Discovering a metabolic pathway linking several amino acids

In this section we analyze the capability of PhDSeeker to build a pathway that relate five amino acids with different properties. For this purpose, we selected threonine (C00188, neutral and polar), methionine (C00073, neutral and nonpolar), phenylalanine (C00079, aromatic), arginine (C00062, basic) and aspartic acid (C00049, acid), and we use the latter as initial substrate. The search was performed using 10 ants, and reactions in the *ecoli* dataset also were specified as the boundary for this search.

**Figure 5:**
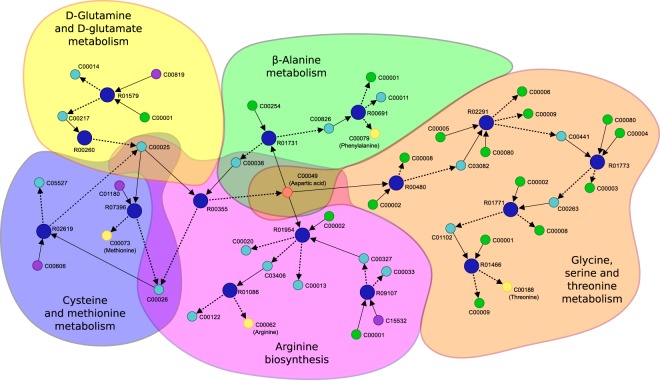
Example of a metabolic pathway linking threonine (C00188, neutral and polar), methionine (C00073, neutral and nonpolar), phenylalanine (C00079, aromatic), arginine (C00062, basic) and aspartic acid (C00049, acid). Well-known pathways involved in the solution are indicated with different colors.

Figure 5 shows an example of a metabolic pathway found and the known pathways to which compounds and reactions belong. Clearly, some compounds participate in several pathways, such as the aspartic acid (C00049) and oxaloacetate (C00036). As it can be appreciated, arginine, threonine and phenylalanine are synthesized by their own pathways, and only share the aspartic acid as initial substrate. This situation is different for methionine, since it can be produced without using aspartic acid, through an independent pathway (reactions in the yellow region) that produce L-glutamate (C00025). This compound together with 4-methylthio-2-oxobutanoate (C01180) are then used to produce methionine. Although aspartic acid does not contribute to produce methionine, it is still related to its synthesis. It is evident that C00025 is a key compound in the synthesis of C00049 and C00073, and must be consumed by R07396 and R00355 to produce their corresponding products. In case of a heavy consumption of C00049, production of this amino acid probably will be preferred, decreasing production of C00073. Fortunately, both reactions produce 2-oxoglutarate (C00026), which is used for reaction R02916 to synthesize more C00025 and continue with the production of methionine. In this context, if R02916 was not present, synthesis of methionine probably would be stopped.

It should be noted here, that this solution comprises several known metabolic pathways, and that the algorithm is clearly able to overcome these limitations and find a feasible pathway that relates all the amino acids. Moreover, this search was performed automatically, saving time and avoiding the need to explore, by hand or text mining, all potential connections among the compounds.

## Conclusion

Synthesizing metabolic pathways is still an open challenge that requires the development of novel and more powerful computational methods. Here, we presented PhDSeeker, a novel ant-based algorithm for synthesizing feasible linear and branched metabolic pathways. Starting from a set of freely available compounds without connections, this algorithm searches for a sequence of feasible reactions that relate a given set of compounds. While explores the solutions space, it expands the original set with new compounds and connections, in order to carry out more reactions. Therefore, each state corresponds to a set of compounds and the relations among them, while transitions between them are performed by applying feasible reactions. This definition leads to a more extensive search space than the one associated to a typical compounds-and-reactions graph. However, our algorithm avoids this problem building solutions while searching and never working on the whole graph. Results show that this algorithm is able to find metabolic pathways linking several compounds, even when considering many compounds and a large number of available reactions. Validation tests demonstrate that this proposal can reproduce well-known pathways and can also synthesize novel solutions. This new algorithm can be a valuable tool for the study of the metabolism, and also for designing novel pathways in metabolic engineering and synthetic biology.

## Source Code Availability

Source code of this algorithm is available at https://sourceforge.net/projects/sourcesinc/files/phdseeker/. Examples for searching metabolic pathways among several compounds are provided. The software is also available as a web demo at http://sinc.unl.edu.ar/web-demo/phdseeker2/.

## Electronic supplementary material

Supplementary Material
