# Supplementary material for: Metabolic pathways synthesis based on ant colony optimization
Source: Sci Rep. 2018 Nov 6;8:16398. doi: 10.1038/s41598-018-34454-z (PMC6219534; doi:10.1038/s41598-018-34454-z)
Supplement: Supplementary file 1 — Supplementary Material [file 41598_2018_34454_MOESM1_ESM.pdf]

Supplementary Material

Metabolic pathways synthesis based on ant colony optimization

Matias F. Gerard, Georgina Stegmayer and Diego H. Milone

Research Institute for Signals, Systems and Computational Intelligence, sınc(i),
FICH–UNL/CONICET, Argentina.

Supplementary Table S1: Datasets used to evaluate the algorithms. Size, composition of the dataset, and reference pathways used to build it are shown.

	number of reactions		
dataset	reversibles	irreversibles	total
glycolysis[a]	25	29	79
proline[b]	24	91	139
xproline[c]	132	179	443
multipaths[d]	192	250	634
ecoli	390	748	1528

[a] http://www.genome.jp/kegg/pathway/map/rn00010.html
[b] http://www.genome.jp/kegg/pathway/map/rn00330.html
[c] KEGG reactions: rn00010, rn00020, rn00030, rn00040 and rn00250
[d] KEGG reactions: rn00010, rn00020, rn00030, rn00050, rn00250 and rn00260

Supplementary Table S2: Abundant compounds employed for searching metabolic pathways. Main cofactors, energy carriers, and some common inorganic compounds are included.

KEGG code	name	KEGG code	name	KEGG code	name	KEGG code	name
C00001	H_2O	C00004	NADH	C00007	O_2	C00010	CoA
C00002	ATP	C00005	NADPH	C00008	ADP	C00080	H^+
C00003	NAD^+	C00006	$NADP^+$	C00009	PO_4^{3-}		

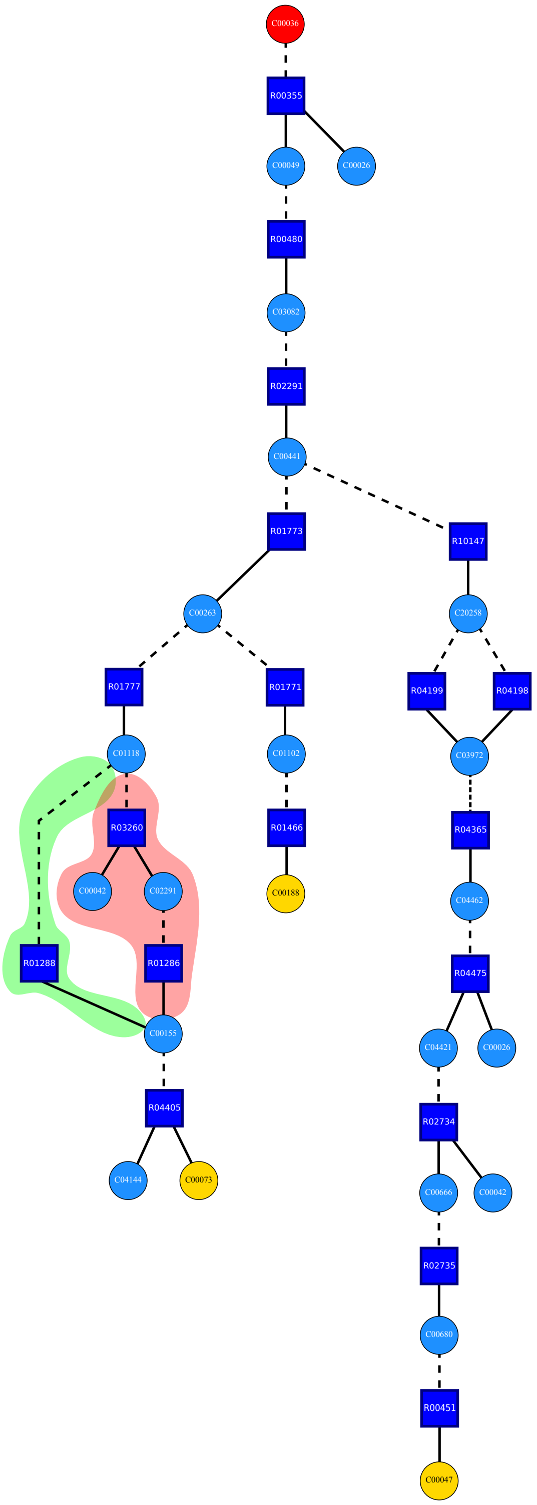

Supplementary Figure S1: standard pathway for the synthesis of of C00047 (L-lysine), C00073 (L-methionine) and C00188 (L-threonine) from C00036 (oxaloacetate). Red circle indicates the initial substrate, yellow circles correspond to final products, and blue squares indicate reactions. Compounds and reactions are indicated in standard KEGG notation. Differences between the standard pathway and the solution found with PhDSeeker are highlighted with colors. Solution found with PhDSeeker replace two reactions of standard pathway (red shading) with only one reaction (green shading), shortening the required number of steps to synthesize C00073.

Parameters sensibility analysis

The effect of the number of ants, evaporation rate (ρ) and α over accuracy and execution time are evaluated. The *superpathway of L-lysine, L-threonine and L-methionine biosynthesis I* (P4-PWY) was taken as a reference pathway to be synthesized. Reactions from EcoCyc v21.5 were used as a list of available reactions to synthesize the reference pathway. Numerical experiments were performed without parallelizing. Accuracy results (Figures S2a and S2b) shows that using 50 and 100 ants, accuracy does not changes with the variation of α and the evaporation rate (ρ). Moreover, it must also be noted that α does not play any important role. Instead, ρ becomes relevant when only a few ants are used, and $\rho \geq 0.1$ allows good accuracy to be obtained. As more ants are used more computation time is required. In summary, it can be stated that few ants (around 10 ants), $\rho \geq 0.1$ and any α can be used to get good accuracy in reasonable times.

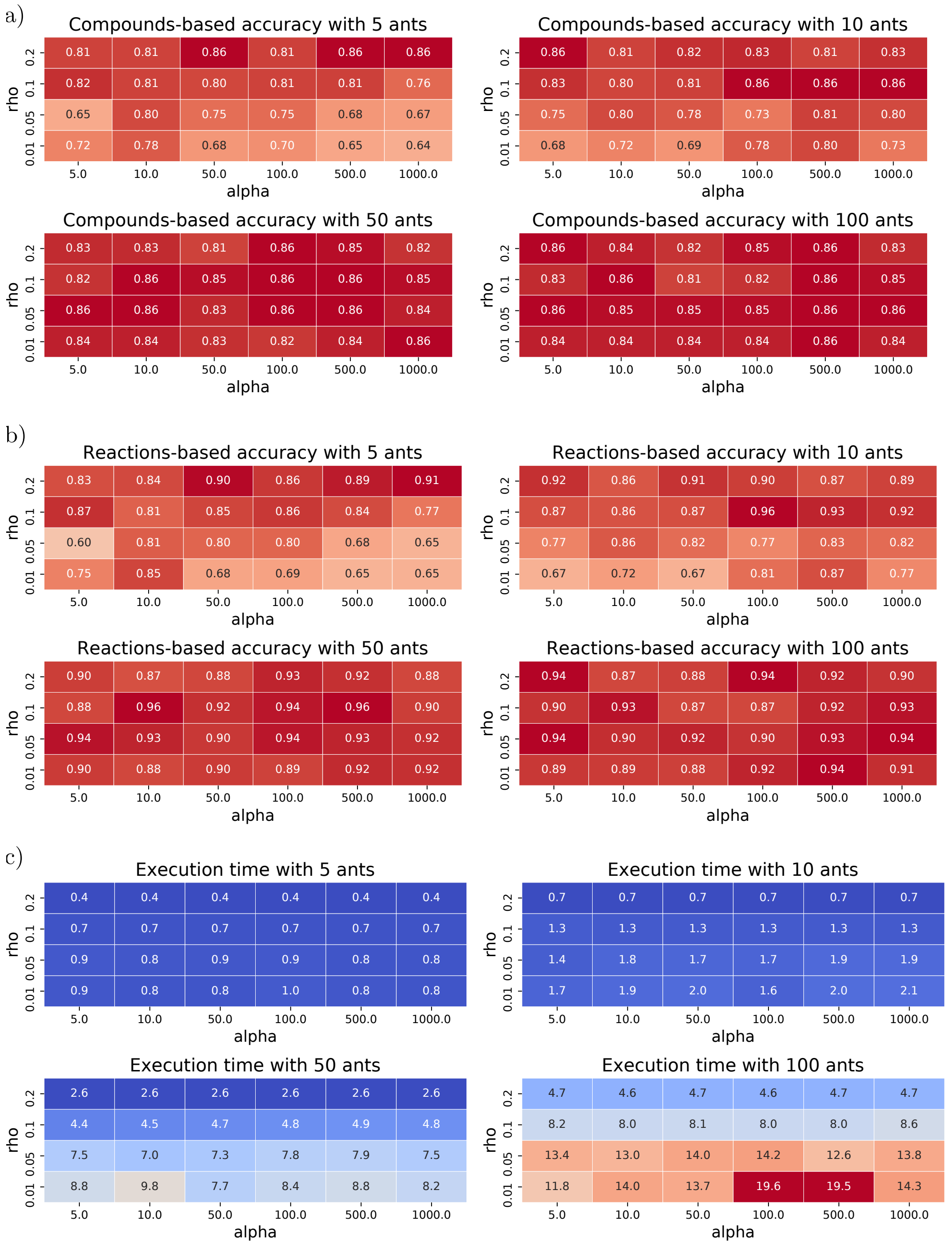

Supplementary Figure S2: PhDSeeker performance versus parameter variation. Every value in the figure corresponds to the median of 10 runs. a) Accuracy calculated taking into account only compounds. b) Accuracy calculated taking into account only reactions. c) Execution time in hours.
